# Quantitative genetics of age-related retinal degeneration: a second F1 intercross between the A/J and C57BL/6 strains

**Published:** 2007-01-25

**Authors:** M. Danciger, H. Yang, R. Ralston, Y. Liu, M. T. Matthes, J. Peirce, M. M. LaVail

**Affiliations:** 1 Department of Biology, Loyola Marymount University, Los Angeles, CA; 2 The Jules Stein Eye Institute, UCLA School of Medicine, Los Angeles, CA; 3 Beckman Vision Center, UCSF School of Medicine, San Francisco, CA; 4 Dept of Anatomy and Neurobiology, University of Tennessee Health Science Center, Memphis, TN

## Abstract

**Purpose:**

Previously, several quantitative trait loci (QTL) that influence age-related retinal degeneration (ageRD) were demonstrated in a cross between the C57BL/6J-c^2J^ and BALB/cByJ strains (B x C). In this study, as a complementary approach to ongoing recombinant progeny testing for the purpose of identifying candidate quantitative trait genes (QTG), a second test cross using the A/J and the pigmented C57BL/6J strains (A x B) was carried out. The albino A/J strain was selected because it had the most amount of ageRD among several inbred strains tested, and the pigmented C57BL/6J strain was selected because along with its coisogenic counterpart C57BL/6J-c^2J^ it had the least amount of ageRD. Thus, the effect of pigment on ageRD could be tested at the same time that the C57BL/6 genetic background was kept in common between the crosses from the two studies for the purpose of comparison.

**Methods:**

A non-reciprocal F1 intercross between the A/J and C57BL/6J strains produced 170 F2 progeny. At 8 months of age after being maintained in relatively dim light, F2 mice, control mice and mice of other strains were evaluated for retinal degeneration by measurement of the thickness of the outer nuclear layer of the retina. The F2 mice were genotyped with dinucleotide repeat markers spanning the genome. Correlation of genotype with phenotype was made with Map Manager QTX software.

**Results:**

Comparison of several strains of mice including the pigmented strains 129S1/SvImJ and C57BL/6J and the albino strains A/J, NZW/LacJ, BALB/cByJ and C57BL/6J-c^2J^, showed significant differences in ageRD. The greatest difference was between the albino A/J strain and the pigmented C57BL/6J strain. However, there was no significant difference between the pigmented C57BL/6J and its albino coisogenic counterpart C57BL/6J-c^2J^. Neither was there significant difference between the pigmented and albino F2 mice from the A x B cross. On the other hand, F2 males had a small but significantly lower amount of ageRD than females. Several QTL were identified in the A x B cross but surprisingly none of the 3 major QTL present in the original B x C cross (Chrs 6, 10, and 16) was present. There were minor QTL on proximal Chr 12 and proximal Chr 14 in common between the two crosses, and the proximal Chr 12 QTL was present in a previous light damage study involving the B and C strains. At least one sex-limited QTL was present on the X chromosome with a peak in a different location from that of a sex-limited QTL in the previous B x C study. In addition, the protective X allele was from the BALB/cByJ strain in the B x C cross and from C57BL/6J in the A x B cross. In both crosses, the C57BL/6J X-chromosome allele was recessive.

**Conclusions:**

Significant differences were observed in ageRD among several inbred strains of mice maintained in relatively dim light. AgeRD was not influenced by pigment but was influenced by gender, albeit to a small degree. The presence of the same QTL in one light-induced and two ageRD studies suggests at least partial commonality in retinal degeneration pathways of different primary cause. However, the three main QTL present in the B x C cross were absent from the A x B cross. This suggests that the genetic determinants responsible for the greater sensitivity to ageRD of BALB/cByJ and A/J relative to C57BL/6J are not the same. This is supported by the presence of sex-limited X-chromosome QTL in the two crosses in which the C57BL/6J allele is protective relative to the A allele and sensitive relative to the C allele. The findings in the two studies of differing allelic relationships of QTG, and differing QTL aid the identification of candidate genes mapping to critical QTL. Identifying natural modifying genes that influence retinal degeneration resulting from any causal pathway, especially those QTG that are protective, will open avenues of study that may lead to broad based therapies for people suffering retinal degenerative diseases.

## Introduction

The mouse retina differs from the human retina in several ways: it lacks a macula and a fovea, the proportion of cones is lower and the total number of photoreceptors is lower, and although the rods are quite similar to human rods, the cones have different light sensitivity pigments such as one that "sees" in the UV range. In addition, the mouse retina is dichromatic while the human retina is trichromatic. Nevertheless, the diseased mouse retina has served as a model for human disease in many studies including the use of spontaneous, transgenic, knock-out and knock-in models for rod degenerative diseases; models of particular aspects of AMD; light damage models; and in the case of this paper, models of age-related retinal degeneration (ageRD) [1-5]. We have found that a gradual, spontaneous ageRD occurs in many strains of mice and differs among strains due to genetic background. Several inbred strains of mice raised in the same vivarium with the same feed under the same dim (2 to 7 ft.-c.) lighting conditions showed significantly different levels of retinal degeneration when measured at eight months (Table 1), and ten and twelve months of age [6]. This led us to ask which genes are involved in age-related retinal degeneration (ageRD), which alleles of these genes retard the process and which alleles make the animal more susceptible to it.

**Table 1:** Mean outer nuclear layer thickness of strains of mice aged to eight months.

**A/J (14)**	**NZW/LacJ (13)**	**129S1/SvImJ (10)**	**BALB/cByJ (29)**	**Balbino (19)**	**Bpig (21)**
28.76±0.68	31.67±1.35	37.76±0.42	39.05±0.28	42.11±0.58	42.85±0.52

"Balbino" represents C57BL/6J-c2J (albino); "Bpig" represents C57BL/6J (pigmented). Numbers in parentheses are sampling sizes. Outer nuclear layer thickness measurements are in μm±SEM.

Previously, we carried out a quantitative genetics study of a test F1 intercross between the albino mouse strains C57BL/6J-c^2J^ (B) and BALB/cByJ (C). In that study, we identified three major quantitative trait loci (QTL) that influenced ageRD on Chrs 6, 10 and 16 as well as several minor QTL on Chrs 8, 12, 13, and 14. The Chrs 6 and 10 loci represented B alleles that slow ageRD (protective), and the Chr 16 QTL a B allele that renders the retina more susceptible to the process [6]. Since QTL circumscribe large areas of a chromosome, it is necessary to fine map the loci in order to more closely identify candidate quantitative trait genes (QTG). One way to accomplish this is to breed the two original strains to produce interval specific congenic strains [7] with a genetic background that is primarily from one parental strain except for regions within the critical QTL interval that carry alleles from the other parental strain, the sizes of which vary by the position of recombinations within the region. Recombinant progeny determined by genotyping across the QTL interval are tested for phenotype (in this case, after eight months) in order to refine the critical area of the QTL to a size containing a small enough number of genes that can be reasonably evaluated for variants. Putative QTG must have allelic differences that can account for the difference in ageRD phenotypes between the two strains and must be verified by breeding protocols or transgenic studies. An additional and complementary approach leading to candidate gene identification more quickly is to breed and phenotype a second test cross with one or two additional strains. Identification of common QTL present in the second cross provides clues about the identity of QTG based on haplotype differences among the strains, and contributes to the verification of a QTG by comparison of alleles among strains. QTL present in one cross and absent in a second can also provide clues to the identities of QTG, assuming that the confirmatory cross has sufficient power to allow detection of the QTL. We report here on a quantitative genetics study of an F1 intercross between the pigmented C57BL/6J strain and the albino A/J strain including haplotype analysis and the influence of strain, pigment and gender on ageRD.

The identification of genes/alleles that influence ageRD will provide candidates for study in the human as susceptibility genes in complex genetic retinal degenerations such as AMD and diabetic retinopathy and as modifiers of variant monogenic RD phenotypes such as *RP1*-caused retinitis pigmentosa [8,9], or *RS1*-caused retinoschisis [10,11].

## Methods

### Mice

A/J, C57BL/6J, BALB/cByJ, 129S1/SvImJ, NZW/LacJ, and C57BL/6J-c^2J^ mice were originally purchased from the Jackson Laboratories (Bar Harbor, ME) and maintained through many generations in our vivarium before study. C57BL/6J-c^2J^ mice are derived from the C57BL/6J strain and differ by virtue of a mutation inactivating the tyrosinase gene (c) thus making the strain albino. Therefore, the strain is coisogenic with the pigmented C57BL/6J strain. By convention, all strains of C57BL/6J mice are abbreviated with "B", BALB/cByJ mice with "C" and A/J mice with "A." All mice were kept under a 12-12 h cyclic light cycle with an in-cage illuminance of 2-7 ft-c. The temperature of the vivarium was maintained between 20 °C and 22 °C. Cages were kept on four shelves of free-standing, double-sided, five-shelf racks (never on the top shelf). Each week, the cages were rotated by shelf, by side of the rack and by position on the shelf (seven positions from side to side). Mice were maintained on a low fat diet (number 15001 Rodent Lab Chow, LabDiet) with chow and water ad libitum.

For the quantitative genetics study, a non-reciprocal (A/J x C57BL/6J)F1 intercross was made and 170 F2 progeny were aged to eight months along with 14 A/J, 14 C57BL/6J, and 31 F1 controls. An additional 7 C57BL/6J were considered for comparison of ageRD with other strains. Since the mothers of all the F1s were A/J, all F1, and F2 mice had the A strain mitochondrial genome and all F1 and F2 males had the B strain Y chromosome.

### Quantitative traits

After aging to eight months, eyes were enucleated from mice immediately after euthanasia by carbon dioxide asphyxiation, fixed in a mixture of 2% formaldehyde and 2.5% glutaraldehyde in phosphate buffer, embedded in a mixture of Epon 812 (EMS, Ft. Washington, PA) and Araldite 502 (Tousimis, Rockville, MD) and bisected along the vertical meridian through the optic nerve head. A single 1 μm section was taken from the cut surface of one of the half-orbs from each mouse and stained with toluidine blue as described previously [12]. On this section, measurements of the thickness of the outer nuclear layer (ONL) were made; three measurements each spaced 50 μm apart were taken at nine 0.25-mm intervals both in the superior and inferior hemispheres starting from the optic nerve head [12]. The means of 54 measurements from the entire retinal section were used to score the mice for the quantitative trait. All procedures involving the mice adhered to the ARVO Resolution on the Use of Animals in Research and the guidelines of the Loyola Marymount University Committee on Animal Research.

### Genotyping

Genotyping included the typing of the F2 progeny for 86 dinucleotide repeat markers with an average spacing across the genome of 13.1 cM and was provided by the Center for Inherited Disease Research (CIDR). CIDR is fully funded through a federal contract from the National Institutes of Health to The Johns Hopkins University, Contract Number N01-HG-65403. For each chromosome, the most proximal marker genotyped was within 15 cM of the centromere, internal markers were no more than 30 cM apart and the most distal markers were within 15 cM of the telomere. The exceptions to the above were: Chr 5 where the most distal marker was 24 cM from the telomere, and Chrs 18 and X where the most proximal markers were 22 and 16 cM from the centromere, respectively. A list of markers that are polymorphic between C57BL/6J and A/J mice is available on the CIDR web site under "Mouse Genotyping Services, STRP Resources." A list of the 86 markers used for this study is available upon request. All map positions were based on the Encyclopedia of the Mouse Genome from the Jackson Laboratories web site Mouse Genome Informatics (MGI). Physical distances were taken from the mouse build 36.1 NCBI database.

### Mouse genomic DNAs

Genomic DNAs were isolated from livers with the Puregene® DNA isolation kit (Gentra systems, Minneapolis, MN).

### Data analysis

Data were analyzed with Map Manager QTX20b [13]. With this program, a likelihood ratio statistic (LRS) correlating genotype with phenotype was listed for each of the 86 marker genotypes with a p<0.05. The one with the highest LRS was analyzed by interval mapping of all the markers on its chromosome. The marker at the peak of this QTL was put into the background for the next evaluation. Then peak markers from both the first and second interval maps were put into the background for the next determination, and so on. The above procedure was followed until the LRS was no longer even suggestive of a QTL To determine significance levels for this genome-wide screen, a test of 500 permutations of all marker genotypes together was performed. LRS were converted to LOD scores by dividing by 4.6 (2x the natural log of 10).

## Results

In a previous study, the thickness of the outer nuclear layer (ONL) of the retina was found to differ significantly between the C57BL/6J-c^2J^ and C strains at eight, ten and twelve months of age but not at two, four and six months [6]. From eight to twelve months the loss of photoreceptors increased at the same rate in both strains [6]. Therefore, we measured the thickness of the ONL in six strains after eight months rearing in dim cyclic light (2-7 ft-c, 12 h cycle) to determine if other strains showed significant differences in ageRD between one another. Table 1 shows the differences among the strains in retinal ONL thickness in ascending order (decreasing ageRD), and Table 2 shows the p values for comparisons between strains. Each strain is significantly different from its nearest neighbor with the exceptions of A/J and NZW (p=0.06) and the C57BL/6J and C57BL/6J-c^2J^ coisogenic strains (p=0.34).

**Table 2:** P scores of comparisons of the mean outer nuclear layer thickness of the retinas of six mouse strains at 8 months.

	**NZW/LacJ**	**129S1/SvImJ**	**BALB/cByJ**	**Balbino**	**Bpig**
A/J	0.06	8.9x10-10	1.0x10-19	9.6x10-16	1.4x10-17
NZW/LacJ		9.9x10-4	4.2x10-9	7.0x10-9	3.1x10-10
129S1/SvImJ			0.02	2.4x10-5	7.9x10-7
BALB/cByJ				3.7x10-6	1.2x10-8
Balbino					0.34

"Balbino" represents C57BL/6J-c2J (albino); "Bpig" represents C57BL/6J (pigmented). Comparison p scores were determined with the Student's t-test.

We selected the A/J strain to cross with the C57BL/6J strain because it showed the most ageRD (relative to the B strain) of all strains tested (Table 1). A small number (170) of F2 progeny mice were bred because we were only interested to determine if the major QTL on Chrs 6, 10 and 16 from the original B x C cross [6] would be present in this cross as well. Since the B strain was in common between the B x C cross and the present A x B cross, we reasoned that if the A and C alleles were the same at these loci, 170 progeny would be more than enough to detect the three highly significant QTL. The QTL identified with the Map Manager QTX program are shown in Table 3. All three major QTL from the original B x C cross [6] were absent from this A x B cross suggesting that the QTG alleles of C and A for the Chrs 6, 10, and 16 QTL are different. On the other hand, the weaker QTL on proximal Chr 14 and proximal Chr 12 were in the same regions in both crosses. Different markers were used at different chromosomal intervals in the two crosses so an exact comparison could not be made. However, in the B x C cross, the peak LOD score of the Chr 14 QTL was between D14Mit126 (cM 5) and D14Mit157 (cM 28) and in the A x B cross between D14Mit98 (cM 3) and D14Mit60 (cM 15). The Chr 12 QTL peak LOD score was between D12Mit60 (cM 16) and D12Mit236 (cM 22) in the B x C cross and between D12Mit59 (cM 13) and D12Mit91 (cM 29) in the A x B cross. In both crosses the proximal Chr 14 B allele was protective and the proximal Chr 12 B allele left the retina more sensitive to ageRD. There were three additional weak QTL on distal Chr 12 and Chrs 13 and 17. Pigment did not influence retinal degeneration in this study but gender did. Thus, while albino and pigmented F2 progeny showed no difference in ONL thickness at eight months, females had a small but significantly greater loss of photoreceptor nuclei (thinner ONL) (Table 4).

**Table 3:** Quantitative trait loci from the age-related retinal degeneration A x B intercross study.

**Sig^1^**	**Marker(s) at peak of QTL**	**cM^2^ from centromere**	**Mb^3^ from centromere**	**LOD score**	**% effect (%total genetic effect)^4^**	**Best fitting inheritance model**
HS	59^5^ controls	---	---	27.76	89 (100)	dominant
Sugg	D12Mit16	53	105.3			
	D12NDS2	59	---	2.91	8 (9)	recessive
Sugg	D13Mit13	35	55.1			
	D13Mit256	40	---	2.85	7 (8)	add/dom^6^
Sugg	D17Mit51	22.9	41			
	D17Mit20	34.3	54.9	2.22	5 (6)	dominant
Sugg	D14Mit98	3	15			
	D14Mit60	15	46	2.15	5 (6)	additive
Sugg	D12Mit59	13	28.1			
	D12Mit91	29	68.6	2.24	-5 (-6)^7^	recessive

Analysis with Map Manager QTXb20. Quantitative trait based on the mean of all outer nuclear layer (ONL) thickness measurements. ^1^HS represents highly significant; S represents significant; Sugg represents suggestive, ^2^cM positions are the distances from the centromere taken from the MGI map, ^3^Mb positions were determined from MGI based on the UniSTS annotation of NCBI Build 36.1 and rounded to the nearest 0.1 million bases. Distances shown are from the acrocentric centromere, ^4^The percent total genetic effect represents percentage effect for this locus divided by total percentage effect of controls (89%) rounded to the nearest whole number, ^5^the 59 controls include 14 A/J, 14 C57BL/6J, and 31 F1 mice, ^6^when two models are noted, both have similar percentage effects and similar LOD scores, ^7^The negative or "-" percentage genetic effect score indicates a B-susceptible allele; all other percentage genetic effect scores indicate B-protective alleles.

**Table 4:** Comparison of age-related retinal degeneration in pigmented versus albino and male versus female mice^1^_._

**Mice**	**Mean ONL in μm±SEM (n)**	**p value from Student's t test**
B x A/J, F2 pigmented	39.75±0.25 (119)	
B x A/J, F2 albino	39.96±0.34 (51)	p=0.670
B x A/J, F2 male	40.48±0.26 (93)	
B x A/J, F2 female	39.06±0.34 (77)	p=8.7x10-4

^1^Mice were aged to 8 months in dim cyclic light.

To determine if any genes were acting together to influence ageRD in a significant, synergistic way, we used the interaction function in Map Manager QTX. For an intercross, this function tests every marker as an additive and dominant allele against every other marker as additive and dominant (four interactions per pair of markers). The interaction likelihood ratio statistic (IX) needed for significance is about 20 (LOD score of 4.35) for an intercross (QTX manual). When this function was performed with an exclusion probability of 10^-5^ (as the manual recommends), no interactions were found.

**Figure 1:**
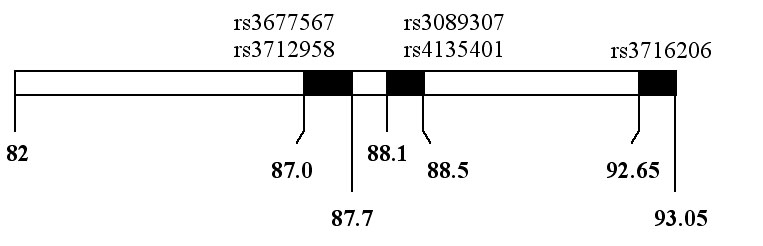
Diagram of the tentative critical region of the age-related retinal degeneration, Chr 6 quantitative trait locus. The three intervals containing SNPs with the genotype A=B not equal to C are shown. Numbers represent megabase distances from the centromere of mouse Chr 6. The names of the SNPs are above each critical region. The last interval extends beyond the distal end of the critical region.

In terms of haplotype, the present A x B cross results suggest that at least the C allele of the Chrs 6, 10, and 16 QTG are not the same as the A alleles; otherwise these QTL would be present in the A x B cross. From the B x C cross, we also know that the B and C alleles of the QTG in these 3 QTL cannot be the same. Therefore, as a means of prioritizing the search for candidate genes, any haplotype regions where the A and C alleles are the same and/or the B and C alleles are the same can be reduced in priority, though not actually eliminated. Considering that we have some preliminary recombinant progeny testing data that tentatively reduces the Chr 6 QTL to 10-11 Mb (data not shown) on the distal side and that the Chrs 10 and 16 QTL are quite broad, we focused on the Chr 6 QTL. We examined a list of SNPs and corresponding genotypes in this critical region kindly provided by Drs. David Delano and Tim Wiltshire from the Genomics Institute of the Novartis Research Foundation, San Diego (GNF). The list was constructed from three sources: GNF [14], Rosetta/Merck [15], and the Broad Institute. After assigning a lower priority to B=C and A=C regions, only 5 SNP's of the 701 SNP's identified in this region were left each with allele characteristics A not equal to C and B not equal to C. These were at about 87.2, 87.5, 88.3 [2] and 92.85 Mb, respectively. We selected regions of about 400 kb with the corresponding SNP (or SNP pair) in the center and searched NCBI MapView for gene candidates. Since the first 2 SNP regions overlapped, the first region was 87.0 to 87.7 Mb, then 88.1 to 88.5 Mb and 92.65 to 93.05 Mb (see Figure 1). Within the three regions, a total of twelve annotated genes were present along with two hypothetical genes and five Riken cDNAs. Of these, six were not expressed in eye and five more were only weakly expressed (Unigene: NCBI). For one hypothetical gene there was no expression data. Six of the seven remaining genes were annotated -*Eefsec, Ruvbl1, Sec61a1, Gfpt1, Antxr1, Prickle2*-and the last was a Riken cDNA, *8430417A20Rik*. The five genes weakly expressed in the eye were *Prokr1*, *Gkn1, Gata2* and two more Riken cDNAs, *2010301N04Rik* and *E230015B07Rik*. Using the WebQTL tool in GeneNetwork [16],  and the MGI mouse SNP QueryForm, we searched all of the above genes for SNPs that fit either of two categories: (1) a difference in alleles between both the B and C strains and the A and C strains or (2) the same alleles for the A and B strains if C strain data was not present. Four SNPs in category (1) and three more in category (2) were found, three in *Antxr* (Anthrax receptor 1), three in *Ruvbl1* (Ruvb-like protein) and one in *Eefsec*; (eukaryotic elongation factor, selenocysteine-tRNA-specific). All of the SNPs were in intronic sequences distant from exons. Each of these three genes was entered into the WebQTL database and queried for trait correlation in eye tissue of BxD (C57BL/6J x DBA/2J) recombinant inbred strains. None of the three genes showed a coordinate expression with retina- or RPE-specific genes. Nevertheless, they remain candidates for further study by virtue of their Chr 6 loci and SNP genotypes.

The Map Manager program cannot analyze X chromosome QTL because it cannot distinguish between hemizygous male and homozygous female genotypes. Therefore, we evaluated the influence of the loci on the X chromosome by other means.

**Figure 2:**
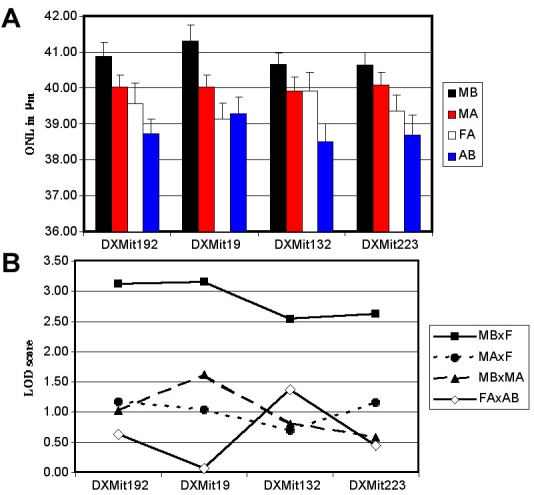
Comparison of average outer nuclear layer thickness scores of F2 progeny by genotype for dinucleotide repeat markers on the X chromosome. MB represents males hemizygous for the B allele; MA represents males hemizygous for the A allele; FA represents females homozygous for the A allele; AB = heterozygous females; F represents all females. **A**: Histogram of mean outer nuclear layer (ONL) thickness±SEM by genotype. **B**: Plots of the negative logarithm of the probability of significant difference determined by the unpaired Student's t-test between mean ONL thickness values of males and females of the same genotype for all four X chromosome markers. The markers are evenly spaced in the figure. The spacing does not represent true cM distances along Chr X.

For each of the four X chromosome markers, we calculated the mean ONL thickness for F2 males hemizygous for the A allele (MA), F2 males hemizygous for the B allele (MB), F2 females homozygous for A (FA) and heterozygous F2 females (AB) (because of the breeding plan, there were no F2 females homozygous for X chromosome B alleles). Figure 2A shows the mean ONLs of the F2 progeny with the various X chromosome marker genotypes. The males hemizygous for B consistently had ONLs thicker than females and males hemizygous for A. To evaluate the significance of this, we performed t-tests comparing mean ONL thickness for the two male and two female F2 genotypes at each marker.

In Figure 2B, we compared all females to males hemizygous for B and to males hemizygous for A since F2 males on average had thicker ONLs than F2 females (Table 4). On the other hand, A/J male and A/J female controls did not differ significantly from each other; neither did C57BL/6J male and female controls nor F1 male and female controls (Table 5). This supported the idea that the F2 difference was not due to gender alone but rather to the presence of a sex-limited QTL. Figure 1B shows that males hemizygous for B were significantly different from females for all 4 markers DXMit192, DXMit19, DXMit132, DXMit223 (LOD scores 3.12, 3.15, 2.54, 2.63, respectively) suggesting that QTG on the B X chromosome provide a measure of sex-limited protection against ageRD relative to the A/J X chromosome. The Chr X QTG B alleles of DXMit192 and DXMit19 at the peaks of the QTL (Figure 2B) are recessive since the heterozygous females (both A and B Chr X alleles present) did not have significantly less degeneration than females of AA Chr X genotypes.

**Table 5:** Mean retinal outer nuclear layer thickness of aged control male and female mice.

	**A/J**	**C57BL/6J**	**F1**
Males	29.71±0.61 (8)	42.61±0.60 (10)	40.42±0.26 (12)
Females	27.50±1.26 (6)	43.07±0.86 (11)	41.24±0.52 (19)
p	0.11	0.68	0.13

Mice were aged to 8 months in dim cyclic light. Retinal outer nuclear layer thickness means are expressed in μm±SEM; numbers of mice are in parentheses.

Genotyping markers for the X Chr were quite similar for the B x C and A x B crosses. The map positions of the 4 markers used in each cross were for the B x C cross 17, 43, 63 and 73 cM (DXMit68, DXMit19, DXMit216, DXMit223) and for this A x B cross 16, 43, 60 and 73 cM (DXMit192, DXMit19, DXMit132, DXMit223). Both crosses were non-reciprocal such that F2 female X-chromosome alleles were either homozygous C or heterozygous in the B x C cross, and homozygous A or heterozygous in the A x B cross. In both cases, alleles of QTG on the X chromosomes influenced ageRD. However, in the B x C study [6] it was the Chr X C alleles (relative to B) and in this study it was the B alleles (relative to A) that offered protection against ageRD. In both cases, all 4 markers showed significant LOD scores ranging from 2.57 to 3.59 in the B x C study when comparing ONL of males with Chr X C alleles with those of all females, and 2.54 to 3.15 in this study when comparing ONL of males with Chr X B alleles with those of all females. The peak LOD scores of the two studies did not match as the third marker (DXMit216 at 63 cM) of the B x C study was at the peak while it was the first and second markers of this A x B study (DXMit192 and DXMit19 at 16 and 43 cM, respectively). Therefore, it is likely that at least two different QTL are present in the two crosses. This is supported by the fact that C protects relative to B in the B x C cross and B protects relative to A in the A x B cross.

## Discussion

**Figure 3:**
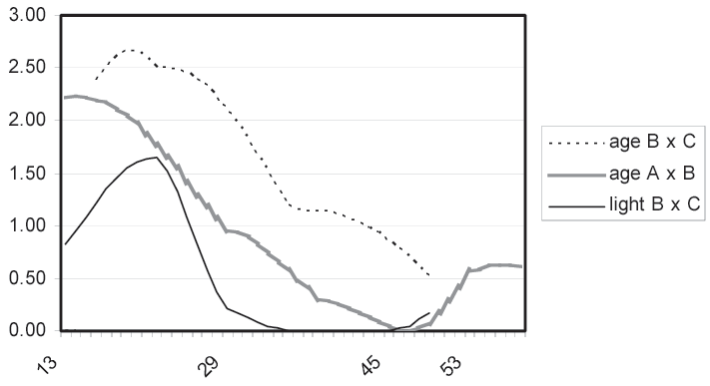
A quantitative trait locus on proximal Chr 12 is present in three different studies. Comparison of proximal Chr 12 quantitative trait loci (QTL) from, the B x C age-related retinal degeneration (ageRD) study [6], this A x B ageRD study and the B x C light damage study [18]. The Y axis is the LOD score and the numbers listed on the X axis are the cM positions on Chr 12.

In a quantitative genetics study of the A/J and C57BL/6J strains of mice, we have identified several QTL on autosomes and one or more significant QTL on the X chromosome that influence ageRD. The study was carried out for the purpose of comparison to a previous ageRD study of the BALB/c and C57BL/6J-c^2J^ strains in which we identified three highly significant QTL on Chrs 6, 10, and 16, respectively. Considering the relatively close strain relationship between A/J and BALB/c and the relatively distant relationship between the two and C57BL/6 [17], it was surprising that none of the three major QTL from the original B x C cross [6] showed up in this cross. It is unlikely that these three QTL were not detected due to the relatively low number of F2 progeny in this cross at 170 (compared to 268 in the B x C cross) because weaker, suggestive QTL on proximal Chr 14 and proximal 12 present in the previous cross were also present in this cross,. Interestingly, the suggestive proximal Chr 12 QTL was also present in a retinal light damage study between the B and C strains [18], suggesting a more fundamental role in retinal degeneration for the corresponding QTG (Figure 3). However, pursuit of the identity of the QTG in the proximal Chr 12 or proximal Chr 14 QTL is prohibitive because of the very small effect of each.

For the Chr X QTL, males hemizygous for B alleles have significantly less ageRD than females with A/J alleles but not significantly less than males hemizygous for A/J alleles. Therefore, it is the combination of Chr X B alleles and male gender that accounts for the significant difference. One explanation is a contribution of the Y chromosome from the B strain. Since the cross was non-reciprocal all F2 mice carried the B strain Y chromosome. Thus, males carrying Chr X B QTL alleles and the B Y chromosome differ in two ways from females carrying Chr X A alleles (or AB but B alleles are recessive) and no Y chromosome, but in only one way from males carrying Chr X A/J QTL alleles because they share the B Y chromosome. However, pursuit of the identity of the QTG in the X chromosome like those of Chrs 12 and 14 is not likely to be successful because of the very small effect of each. It is more feasible to search for the QTG of the strong QTL on Chrs 6, 10 and 16 which were observed in the B x C study. We focused on the Chr 6 QTL because we have done some recombinant progeny testing and have tentatively reduced the critical interval to 10-11 Mb (data not shown).

Three Chr 6 QTL ageRD candidate genes were identified on the basis of SNP analysis and location, *Antxr1*, *Ruvbl1* and *Eefsec*. The Human orthologues of these three genes are *ANTXR1* (Anthrax receptor 1, a type I transmebrane protein), *RUVBL* (Ruvb-like protein, a TATA binding protein interacting protein), and *EEFSEC* (eukaryotic elongation factor, selenocysteine-tRNA-specific). Although we have not found a specific association with retinal or RPE function for any of these three genes, all three are expressed in the eye (based on the corresponding expression profiles for each of these genes listed in Unigene). The SNP inclusion criteria for the Chr 6 QTL were that the A allele did not equal the C allele and the B allele did not equal the C allele. This was because the B strain was common to the two intercrosses and the Chr 6 QTL was present in the B x C cross (B does not equal C) and absent from the A x B cross (A does not equal C). It is important to note that SNPs fitting these criteria could be present that were not noted/detected in the various databases and maps since new variations can occur at any time and/or the density of markers in a region may not be sufficient to identify distinguishing markers that are present but have not been detected. Nevertheless, any Chr 6 QTL ageRD candidate gene that has a significant variant that differs between the B and C strains and also differs between the A and C strains would be prioritized for study. Such a candidate gene would encourage further studies to verify its function and would open additional avenues of study leading to a greater understanding of retinal degenerative diseases.
